# Irisin – a myth rather than an exercise-inducible myokine

**DOI:** 10.1038/srep08889

**Published:** 2015-03-09

**Authors:** Elke Albrecht, Frode Norheim, Bernd Thiede, Torgeir Holen, Tomoo Ohashi, Lisa Schering, Sindre Lee, Julia Brenmoehl, Selina Thomas, Christian A. Drevon, Harold P. Erickson, Steffen Maak

**Affiliations:** 1 Institute for Muscle Biology and Growth, Leibniz Institute for Farm Animal Biology, D-18196 Dummerstorf, Germany; 2 Department of Nutrition, Institute of Basic Medical Sciences, University of Oslo, 0317 Oslo, Norway; 3 The Biotechnology Centre of Oslo, University of Oslo, 0317 Oslo, Norway; 4 Department of Cell Biology, Duke University, Durham, NC 27710, USA; 5 Institute of Genome Biology, Leibniz Institute for Farm Animal Biology, D-18196 Dummerstorf, Germany; 6 Swiss Institute of Equine Medicine (ISME), Vetsuisse Faculty, University of Berne, 1580 Avenches, Switzerland

## Abstract

The myokine irisin is supposed to be cleaved from a transmembrane precursor, *FNDC5* (fibronectin type III domain containing 5), and to mediate beneficial effects of exercise on human metabolism. However, evidence for irisin circulating in blood is largely based on commercial ELISA kits which are based on polyclonal antibodies (pAbs) not previously tested for cross-reacting serum proteins. We have analyzed four commercial pAbs by Western blotting, which revealed prominent cross-reactivity with non-specific proteins in human and animal sera. Using recombinant glycosylated and non-glycosylated irisin as positive controls, we found no immune-reactive bands of the expected size in any biological samples. A *FNDC5* signature was identified at ~20 kDa by mass spectrometry in human serum but was not detected by the commercial pAbs tested. Our results call into question all previous data obtained with commercial ELISA kits for irisin, and provide evidence against a physiological role for irisin in humans and other species.

In 2012, Boström *et al.*^1^ described irisin as a cleaved and secreted part of the transmembrane protein *FNDC5* (fibronectin type III domain containing 5). They proposed irisin as an exercise-induced myokine triggering “browning” of white adipose tissue. These findings sparked a debate mainly turning on two issues: (I) the relevance of irisin in humans, (II) the specificity of commercially available enzyme-linked immunosorbent assays (ELISA), and more specifically the polyclonal antibodies on which they were based.

First, following the initial study^1^, it was realized that the start codon of the human *FNDC5* gene is mutated from the normal ATG to ATA. There are examples of proteins being expressed from unusual start codons^2^, however, Raschke *et al.*^3^ found that *FNDC5* transcripts derived from the AUA start codon were translated to protein with extremely low efficiency as compared to the normal AUG start codon. All other animal species have an ATG as start codon at this position. This suggests that the human species has an effective gene knockout of *FNDC5* and, consequently of irisin. Furthermore, Timmons *et al.*^4^ expressed doubts about the response of *FNDC5* mRNA in human muscle to exercise, based on their previous and larger data sets, which showed no such response. Nevertheless, a number of research groups around the world have examined the effects of exercise on irisin levels in human serum. These studies, mostly using commercial ELISA kits that are questioned here, have given contradictory results. Huh *et al.*^5^ observed a significant increase in serum irisin in response to acute exercise after 1 week of moderate training whereas no effect of acute exercise was found after 8 weeks of training intervention. Short term effects of exercise on irisin levels were also reported by some authors^5^,^6^,^7^,^8^,^9^. In contrast, no systematic effects of exercise on circulating irisin were found in several other studies^10^,^11^,^12^,^13^,^14^,^15^,^16^,^17^.

Second, the *FNDC5* antibody used in the initial study^1^ was raised against the C-terminus of the protein (amino acids [aa] 149–178), which is not part of the cleaved irisin peptide (aa 32–143; GenPept accession number NP_715637). Thus, as initially noted by Erickson^18^, the 20 kDa band detected in western blots in that study should not be irisin, but is probably a non-specific cross-reacting protein. Further studies employed western blots with different antibodies against this epitope and found immune-reactive bands in the range of 20–26 kDa in serum or plasma of rats, mice and humans^19^,^20^,^21^,^22^. Again, all these antibodies were generated against the C-terminal *FNDC5* segment, which is not part of circulating irisin. An antibody raised against partial irisin (aa 42–112), which should detect irisin, stained a band at 25 kDa as well as bands of higher molecular weight in western blots of the secretome of cultured rat muscle cells and adipocytes^21^. In previous studies, we used an antibody against full-length irisin (aa 32–143) and observed an immune-reactive band at ~13 kDa, the theoretical size of non-glycosylated irisin, in murine serum but not in bovine plasma^23^,^24^.

The therapeutic potential of irisin to fight obesity and diabetes has aroused extensive interest. Several commercial sources have marketed kits for ELISA, EIA, and RIA to detect and quantify irisin in different biological fluids, under different exercise interventions and/or in different diseases (reviewed by Sanchis-Gomar *et al.*^25^, Elsen *et al.*^26^). A striking feature of these investigations was the vast variation of reported irisin levels, differing by orders of magnitude even in healthy subjects. This was the case for results obtained with tests from different manufacturers but also with the same test from a single supplier used in different laboratories. More than 80 studies have been published with irisin levels ranging between 0.01 and more than 2,000 ng/mL in human serum or plasma. These commercial kits were usually tested for the ability to detect bacterially expressed irisin (recombinant protein produced in the bacterial cytoplasm, and therefore not glycosylated), and a wide range of sensitivity was reported. For example, a polyclonal antibody used in an ELISA kit (designated here pAb-A) was reported by the supplier to have a detection range of 0.38–205 ng/mL, and a linear range of 4–41 ng/mL. We believe this is an acceptable validation of the sensitivity of the ELISA kit for assaying purified recombinant irisin, in the absence of other proteins.

However, in addition to recognizing the specific antigen, polyclonal antibodies often cross-react with non-specific proteins. When an ELISA kit, based on a polyclonal antibody against irisin, is used to assay a complex protein sample such as plasma or serum, the signal will include that from any irisin but also that from cross-reacting proteins. Some of these may be present at high levels and can dominate the signal. To control for this, it is necessary to validate the antibody by western blot in the target tissue or fluid. Western blots were shown in the original study^1^, but these showed multiple strong cross-reacting proteins and were based on an inappropriate polyclonal antibody^18^. Only a few of the subsequent studies have presented full-size western blots that reveal cross-reacting proteins in plasma or serum^21^,^23^,^24^,^27^. Many studies using only ELISA have apparently ignored the possibility of cross-reacting proteins contributing to the signal.

In our present study, we examined the specificity and sensitivity of currently available irisin antibodies by comparative western blot analysis. For standards we produced recombinant non-glycosylated irisin (rNG-irisin), which runs on SDS-PAGE as a sharp band at 13 kDa, and recombinant, glycosylated irisin (rG-irisin), expressed in mammalian HEK293 cells and running as a smeared band at 18–25 kDa, with a peak at about 20 kDa. By diluting rNG-irisin into plasma and running western blots we achieved two calibrations: determination of the sensitivity of the assay for irisin, and detection of cross-reacting proteins of various molecular weights. We then extended the assay to survey a range of animal species for the presence of irisin in serum or plasma. In addition, we analyzed samples from a previously published training intervention study^13^ with an additional ELISA and western blots to compare irisin levels obtained with different methods. Mass spectrometry was used to search for *FNDC5* or irisin signatures in human serum at different sizes after SDS-PAGE. Finally, RNA sequencing was employed to gain insight about the abundance of different transcripts of *FNDC5* in human muscle.

## Results

### Detection of rNG-irisin with pAb-A

**Figure 1:**
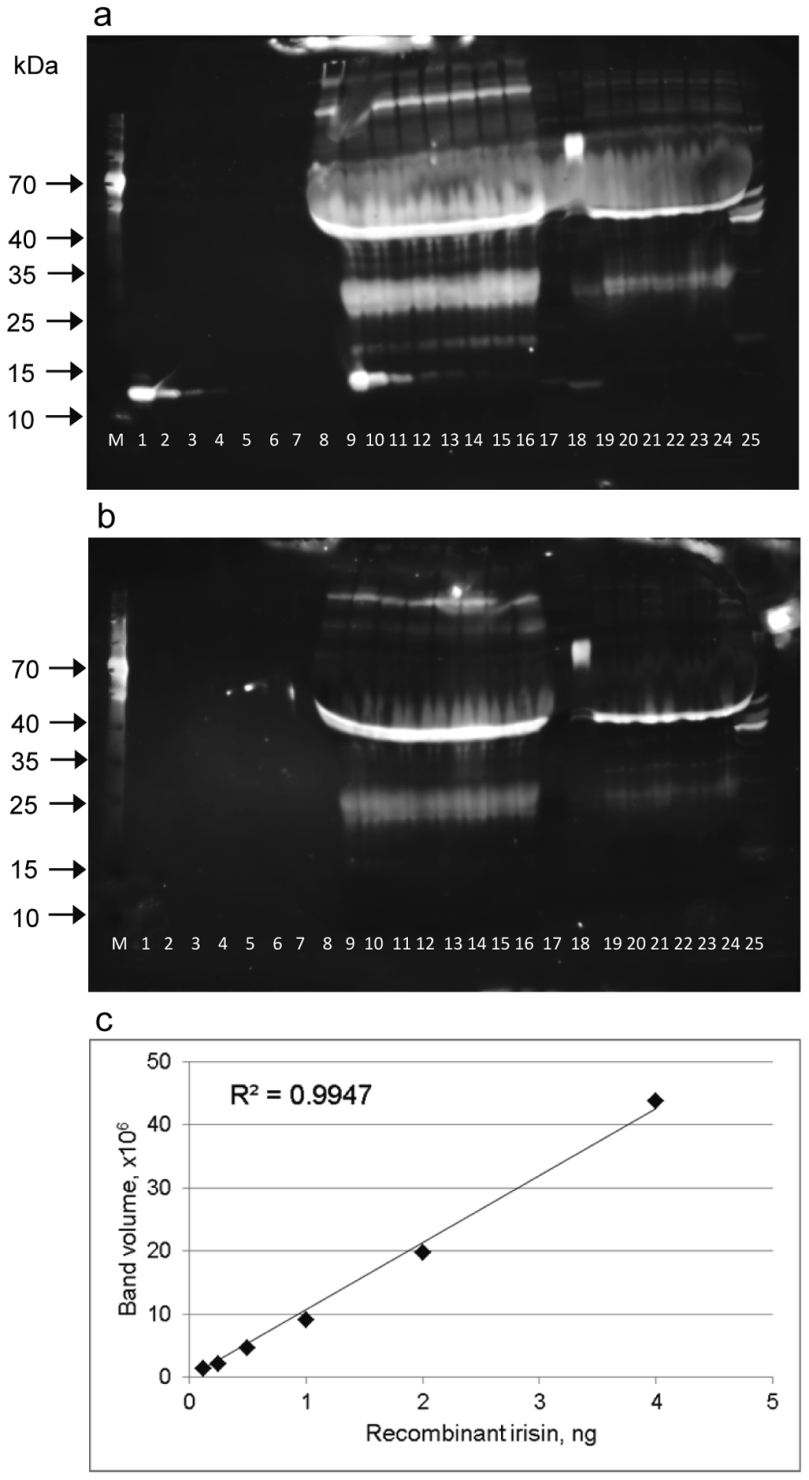
Western blot of dilution series of rNG-irisin, using pAb-A. (a) rNG-irisin was diluted into PBS (lanes 1–8; absolute amounts 20, 4, 2, 1, 0.5, 0.25, 0.125, 0.0625 ng irisin/lane) or diluted in bovine plasma (lanes 9–16; identical amounts). Two samples of mouse serum (lanes 17, 18) and samples of human serum (lanes 19–24) are included. Mouse muscle protein extract is in lane 25. (b) pAb-A was blocked with 5-fold amount rNG-irisin prior to staining the blot. Images were taken after 2 (a) and 4 (b) min exposure to adjust for different background chemiluminescence, and were equally enhanced in contrast. (c) Band volumes of recombinant NG-irisin (lanes 9–15 in (a)) were densitometrically analyzed and plotted against irisin amount (ng) in bovine serum. The highest (20 ng) and lowest (0.0625 ng) concentrations were omitted from this analysis.

Dilution series of rNG-irisin into either phosphate buffered saline (PBS) or bovine plasma were analyzed with anti-irisin pAb-A, raised against full length NG-irisin (Fig. 1a). Bovine plasma was used for the initial test because our previous study had shown no detectable circulating irisin^24^. Two murine sera with unknown irisin levels, human serum samples with irisin levels previously measured with a corresponding ELISA kit (based on pAb-A), and a murine muscle sample were analyzed on the same blot. The antibody reacted with a single band at ~13 kDa in PBS and bovine plasma containing the higher concentrations of added rNG-irisin (Fig. 1a). This band could be completely quenched by preincubation of the primary antibody with 5-fold the amount of rNG-irisin (Fig. 1b). Densitometric analysis of the irisin dilution in bovine plasma revealed linearity in the range from 4 to 0.125 ng irisin/lane (lanes 10–15 in Fig. 1a; R^2^ = 0.9947, Fig. 1c). Recovery rates for the spiked irisin ranged from 75% to 96% in the lanes used for determination of linearity. Because the sample contained 1 μL of plasma we were able to detect irisin at a concentration of 125 ng/mL with this western blot.

The western blot showed several cross-reacting proteins staining much more intensely than even the higher concentrations of added NG-irisin. The band at ~45 kDa is approximately the size for IgG. However, cross-reaction of IgG with the secondary detection system can be excluded because the TrueBlot® system used here is designed to avoid reaction with denatured IgG, and control blots omitting the primary antibody showed no unspecific staining. The band at ~30 kDa is too large to be irisin. The smear from 50–70 kDa and the higher molecular weight bands include albumin and unknown proteins. However, it is clear that these cross-reacting proteins would dominate the signal if this pAb-A is used to assay bovine plasma or human serum.

Notably, irisin was not detected by pAb-A in human serum although levels between 324 and 864 ng/mL were previously reported using the corresponding ELISA kit^13^. This is equivalent to absolute amounts between 0.324 and 0.864 ng/lane on the blot (Fig. 1a), clearly in the linear range of detection by our western blot. A weak band slightly smaller than rNG-irisin was visible in murine serum, but as discussed below is apparently not irisin.

**Figure 2:**
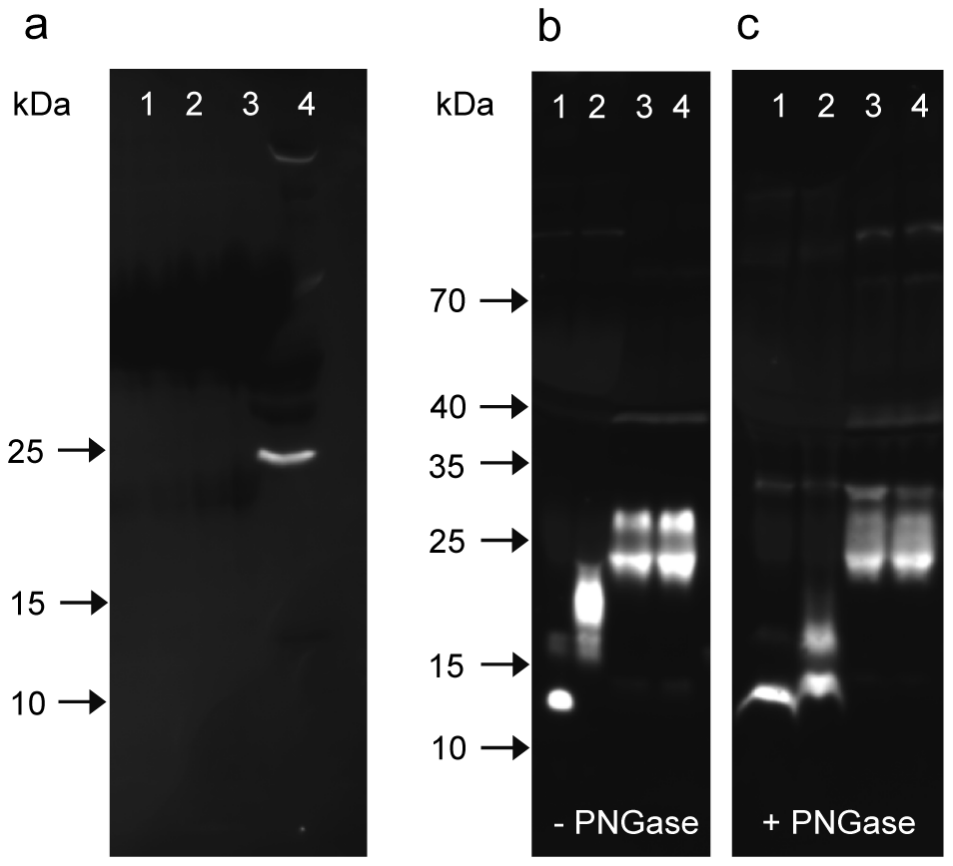
Western blots for detection of FNDC5 and irisin. (a) pAb-E, which reacts with a C-terminal peptide (outside the irisin domain) was used to detect full-length *FNDC5*. This pAb-E detected no band in human serum samples (lanes 1–3), but mouse muscle extract showed a sharp band at ~25 kDa.(lane 4). (b, c) pAb-C was used to stain rNG-irisin (lane 1), G-irisin (lane 2) and human serum samples (lanes 3, 4) before (b) and after (c) deglycosylation with PNGase. All images were taken after 10 min of exposure without contrast enhancement.

The experiment was repeated with antibodies pAb-B, C, and D (Supplementary Fig. 1). All antibodies proved capable of detecting rNG-irisin added to PBS or bovine plasma in a similar linear range. However, none of them stained a band of the expected size for glycosylated irisin in murine or human samples. A strong immune-reactive band at ~25 kDa was observed only with pAb-C in human but not in murine samples which is the upper size limit for glycosylated irisin. However, this band was not detected by any other irisin antibody, thus it is probably a cross-reaction specific for pAb-C. Analysis of serum samples with pAb-E (raised against aa 149–178 of *FNDC5*) revealed no specific band in serum but in murine muscle extract it stained a sharp, single band with the size of full length *FNDC5* (~24 kDa; Fig. 2a). The strong doublet band at about 25 kDa stained uniquely by pAb-C (Fig. 2b) was tested for deglycosylation. In contrast to rG-irisin, whose size was significantly reduced by PNGase, the ~25 kDa doublet was unaffected (Fig. 2b,c). This band was analyzed by mass spectrometry, described later.

Besides the variability in band patterns observed between the tested antibodies, a high lot-to-lot variability was observed for antibodies pAb-A and C (Supplementary Fig. 2). Different lots of antibodies pAb-B and D were not compared.

### Western blot analysis of irisin in serum and plasma of mammalian species

**Figure 3:**
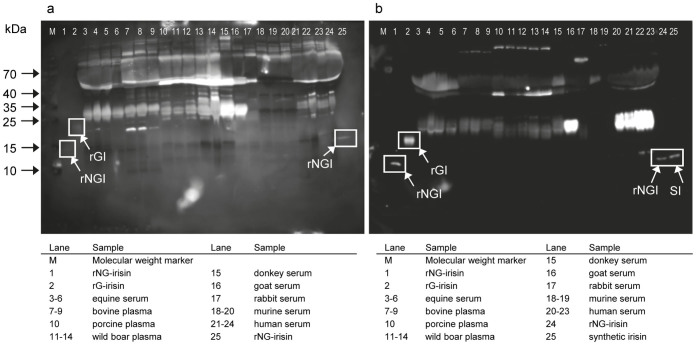
Western blot of serum or plasma samples of different species with irisin antibodies pAb-A (a) and pAb-C (b). Images were taken after 10 min (a) and 20 min (b) exposure and equally enhanced in contrast. Boxes indicate bands of rNG-irisin (rNGI), rG-irisin (rGI), and synthetic (SI) irisin. Samples in lanes 20–25 differ between (a) and (b).

Sera were analyzed from 3 horses undergoing extreme physical exercise (160 km endurance race) and from 1 horse with established metabolic syndrome. For comparison, samples from cattle, domestic pig, wild boar, donkey, goat, rabbit and mouse were included (Fig. 3). Human samples with irisin levels of 76 ng/mL and 864 ng/mL, previously measured with ELISA (pAb-A), were from 1 healthy and 1 pre-diabetic individual prior to or 2 hours after a single bout of acute exercise^13^. All samples were analyzed by western blot with antibodies pAb-A and pAb-C (Fig. 3a,b). Recombinant NG- and rG-irisin were used as positive controls. No antibody detected proteins at the size of the positive controls, indicating that neither non-glycosylated nor glycosylated irisin circulated in the serum/plasma of any species. However, immune-reactive bands were observed at ~16 kDa in a human sample and at ~25 kDa in both human and goat samples with pAb-C. These bands were consequently further analyzed by HPLC/mass spectrometry.

Separate analysis of 11 samples from 6 horses with pAb-C showed a similar band pattern to that in lanes 3–6 (Fig. 3b). Band volumes were not related to physical activity or disease and were not correlated to results obtained with pAb-B on the same samples (Supplementary Fig. 3).

### Comparison of ELISA data with semi-quantitative western blot analyses

**Figure 4:**
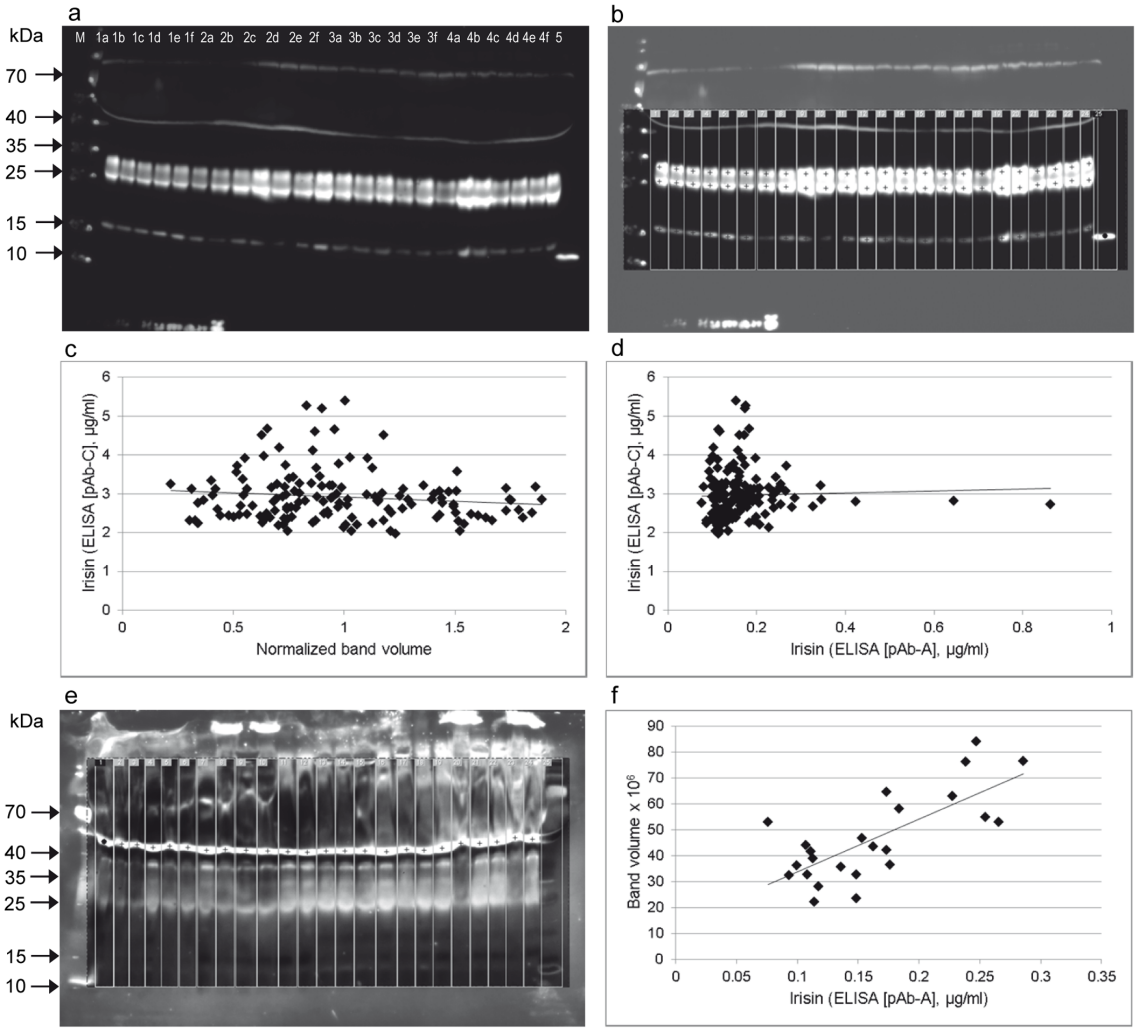
Semi-quantitative analysis of western blots with pAb-C and comparison with ELISA data (based on pAb-A and pAb-C). (a) Western blot (pAb-C) of serum samples of 4 individuals (lanes 1–4) at 6 time points (lanes a–f). RNG-irisin was included as positive control (lane 5). The image was taken after 10 min exposure. (b) Quantification of chemiluminescence of targeted bands (crosses) in marked lanes. Note that contrast enhancement in the region of measurement is only for visualization and does not influence the results. This measurement was done in all 156 samples from 26 individuals of a previous study^13^. (c) Plot of normalized, combined band volumes against irisin levels measured with ELISA based on pAb-C. (d) Plot of irisin levels measured with ELISA based on pAb-A against ELISA based on pAb-C. (e) Quantification of a band (crosses) at 50 kDa detected by western blot of human serum samples with pAB-A. Samples were arbitrarily chosen from the previous study^13^. (f) Volume of the non-specific 50 kDa band was plotted against irisin concentration measured with ELISA based on pAb-A.

A total of 156 serum samples from 26 individuals from a previously published exercise study^13^ were re-analyzed with an additional irisin ELISA based on pAb-C and by semi-quantitative western blot analysis with antibody pAb-C. Irisin levels were previously measured with ELISA based on pAb-A and the data were used for correlation analyses with the results of the present study. Western blot analyses revealed 3 prominent bands at ~16 kDa (larger than rNG-irisin), ~25 kDa, and ~30 kDa in all samples (Fig. 4a). The 16 kDa band was not observed in previous tests with pAb-A (Fig. 1a). Regions of interest were selected and individual band volumes were measured densitometrically (Fig. 4b). There was no significant correlation between normalized volumes of any of the 3 bands or their combined volumes and irisin concentrations measured with the corresponding ELISA kit (pAb-C; r = −0.109, p = 0.46; Fig. 4c). Furthermore, irisin levels measured with the 2 different ELISA kits differed by a factor 18 on average (overall means 2,961 ng/mL vs. 164.5 ng/mL) and were not correlated at all (r = 0.034, p = 0.67; Fig. 4d). In contrast, the volume of a non-specific band observed at ~50 kDa with pAb-A (Fig. 4e) was significantly correlated to irisin levels measured with the corresponding ELISA (pAb-A) in an arbitrarily selected subset of samples (n = 24, r = 0.730, p < 0.001; Fig. 4f). Thus, using the ELISA based on pAb-C (Supplementary Fig. 4) we were unable to repeat the previous finding of an acute effect of exercise on irisin levels in serum, which used an ELISA based on pAb-A^13^.

### Immuno-precipitation and protein identification by mass spectrometry

**Figure 5:**
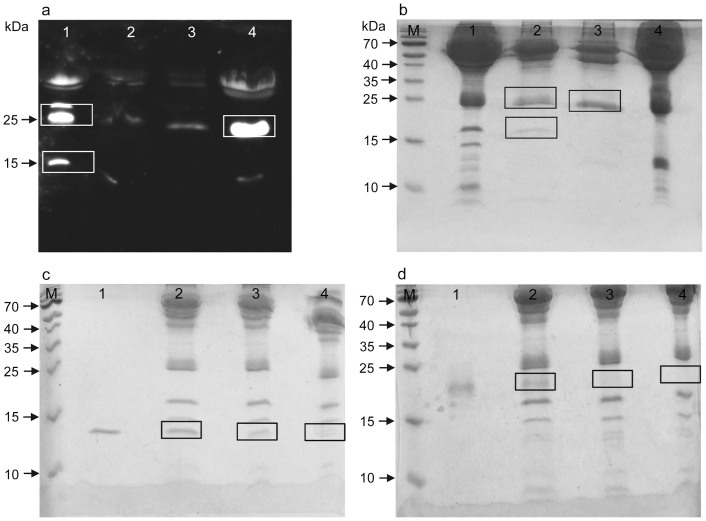
Identification of proteins by mass spectrometry. (a) Western blot of human and caprine serum before (lanes 1, 4) and after (lanes 2, 3) immuno-precipitation with pAb-C. Bands of interest are within white boxes. (b) Coomassie-stained gel with the same samples. After immuno-precipitation, target bands (black boxes) were cut out of the gel and analyzed. (c) Coomassie-stained gel with 500 ng rNG-irisin in PBS (lane 1), 500 ng and 100 ng rNG-irisin added to human serum (lanes 2 and 3) and human serum without addition (lane 4). Serum samples were albumin-depleted prior to electrophoresis. Gel pieces within black boxes were cut out and analyzed. (d) Addition of rG-irisin instead of rNG-irisin. Samples and procedure are as in (c). M: molecular weight marker.

The immuno-reactive bands at ~25 kDa stained by pAb-C in human (Supplementary Fig. 1b, Fig. 3b) and goat serum (Fig. 3b) were purified by immuno-precipitation with pAb-C cross-linked to magnetic beads (Fig. 5a,b). An additional band at ~16 kDa observed earlier in human serum (Fig. 3b) was also included in subsequent mass spectrometric analysis. No peptide corresponding to *FNDC5* or its irisin-part was identified in any of the precipitated samples in 2 repeats of the experiment. Instead, apolipoprotein A1 was identified as a predominant protein in both human and goat bands. The human band of lower molecular weight included mainly Ig kappa chains and hemoglobin subunits.

**Figure 6:**
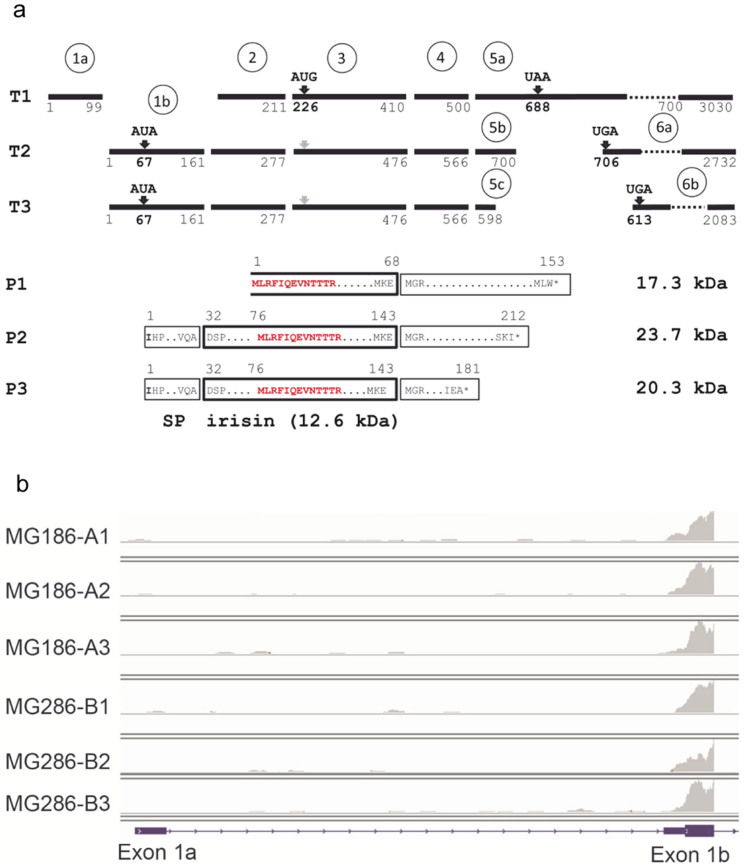
*FNDC5* transcripts and deduced peptides. (a) Transcript structure of human *FNDC5* (T1: NM_001171941.2, T2: NM_153756.2, T3: NM_001171940.1) and deduced peptide structure (P1–P3). Numbers refer to nucleotides (T1-3) or amino acids (P1–3). Black bars represent exons. Exon numbers are given in circles. Start and stop codons are indicated above the bars. The irisin peptide is marked by a bold box. The open box marks the truncated irisin peptide in P1 theoretically resulting from transcript T1. The size of irisin and *FNDC5* protein variants is given. The peptide signature identified by mass spectrometry is marked in red. SP: signal peptide. (b) Example for detection of *FNDC5* transcripts by RNA-sequencing of skeletal muscle biopsies. Exon 1a is specific for transcript T1 whereas exon 1b is part of transcripts T2 and T3. The panels represent results for one individual before (A1–A3) and after 12 weeks of training intervention (B1–B3). A1 and B1 were measured before, A2 and B2 immediately after, and A3 and B3 2 hours after acute exercise^13^.

We then spiked rNG- and rG-irisin into human sera and analyzed bands matched to the size of the recombinant control peptides by HPLC/mass spectrometry (Fig. 5c,d). Samples of human serum without addition of rNG- or rG-irisin were applied to the same gels. Gel pieces at the positions corresponding to the size of the controls were cut out and analyzed by HPLC/mass spectrometry even though no staining with Coomassie Blue was observed. Unique peptide signatures for *FNDC5* or irisin (MLRFIQEVNTTTR or FIQEVNTTTR, Fig. 6a) were detected in samples with added 100 ng or 500 ng rNG-irisin in bands at ~13 kDa. The doublet band visible in human serum without addition (lane 4 in Fig. 5c) contained no *FNDC5* or irisin. Specific signatures for *FNDC5* or irisin were found at ~20 kDa in human serum with 100 ng or 500 ng rG-irisin added. The respective gel piece from a serum sample without added rG-irisin (lane 4 in Fig. 5d) revealed no visible band but contained a single peptide signature unique for *FNDC5* or irisin (FIQEVNTTTR) among 13 other proteins. Besides this, the results from mass spectrometry supported the assumption of massive non-specific reaction of proteins with irisin antibody pAb-C.

### Expression of FNDC5 mRNA in human skeletal muscle

Re-analysis of samples of *m. vastus lateralis* from control and pre-diabetic subjects in a training intervention study by RNA-sequencing confirmed a small but statistically significant increase of total *FNDC5* mRNA levels after 12 weeks of exercise compared to baseline levels (fold change = 1.185, p < 0.001). Effects of the health status were not observed in accordance to the previous analysis^13^.

The current NCBI RefSeq model of the human *FNDC5* gene predicts 3 transcripts (T1: NM_001171941.2, T2: NM_153756.2, T3: NM_001171940.1). Transcript 1 is proposed to be produced by alternative splicing from an upstream exon 1a, whereas the two other transcripts share exon 1b (Fig. 6a). Transcript 1 would produce a truncated *FNDC5* protein from an in-frame AUG codon in exon 3, and the proposed irisin peptide would miss the first 44 amino acids. However, the Kozak sequence around this AUG is very weak and there are 3 other upstream out-of-frame AUG codons that would make translation of this truncated protein unlikely. Thus, analysis of 154 muscle biopsies in the training intervention study^13^ revealed no expression of exon 1a specific for this transcript. In contrast, transcripts 2 and 3 were expressed at high levels as indicated by the data of their shared exon 1b (Fig. 6b). A dissection of the expression to specific transcripts 2 or 3 is not possible due to the structure of the locus (Fig. 6a). All investigated 26 individuals possessed the non-canonical AUA start codon.

## Discussion

Almost 3 years after the discovery of irisin^1^ the debate over its potential role in human metabolism has not been settled. Only recently, Elsen *et al.*^28^ noted that the role of irisin in humans appears still questionable despite the numerous studies. Spiegelman & Wrann^29^ commented in reply that there are more than 45 articles reporting human irisin levels measured with a variety of immunoassays with independently derived antibodies and thus they considered the existence of human irisin in blood a closed issue. However, not one of those studies has addressed the possibility that the immunoassays might be reporting cross-reacting proteins in serum or plasma, and not irisin itself.

Our present study examined 4 antibodies of which 3 were used in corresponding ELISAs. More than 80 studies have been published based on these assays. We showed that all antibodies had prominent cross-reactions with non-irisin proteins in serum or plasma of different species. For one antibody we found a strong correlation between the volume of a prominently stained ~50 kDa band in human serum, which is clearly not irisin, and irisin levels measured with the corresponding ELISA. In contrast, no correlation was found between irisin levels measured with 2 different ELISA kits. Earlier comparisons of different ELISA kits reported only weak correlations and considerable differences in absolute values^12^,^30^. Our data indicate that all previously published assays based on commercial ELISAs, including the more than 45 articles mentioned by Spiegelman & Wrann^29^, were reporting unknown cross-reacting proteins.

Importantly, our Western blots failed to detect irisin or glycosylated irisin in plasma or serum of human and several animal species, even after extreme exercise in the case of horses. A 13 kDa band in mouse serum, identified previously as potential irisin^23^, was stained by pAb-A, but not by -B, -C or -D. This strongly suggests that it is also a cross-reacting protein specific to pAb-A. The detection limit of the most sensitive western blots was about 100 ng/mL being a potential limitation of this study. However, this corresponds to approximately 10 nM which is below the concentration of recombinant *FNDC5* or irisin (20 nM) used in cell culture assays^1^,^31^. Because hormones are typically present in circulation at pM to nM concentrations, it cannot be ruled out that circulating irisin at less than 10 nM could have activity *in vivo*.

Our data on *FNDC5* expression in skeletal muscle demonstrate that the human gene is exclusively translated from transcripts with a mutant start codon. Considering the translation efficiency of such transcripts – 1% compared to transcripts with a canonical start codon^3^ – very low amounts of *FNDC5* could be expected in skeletal muscle. Hence, it is unlikely that irisin can be cleaved and secreted into circulation at amounts measured with ELISAs previously. This is further supported by the observation that acute exercise did not cause a significant up-regulation of *FNDC5* and that the mRNA abundance increased only slightly after 12 weeks of training intervention.

In contrast to most studies, which have neglected the possibility of cross-reaction, a recent study^22^ employed western blots of human serum as the first step. These blots showed several strong cross-reacting bands of higher molecular weight, but the study focused on bands of 32 and 24 kDa, interpreting them as glycosylated and deglycosylated irisin. As shown in our present report the molecular weights of purified rG- and rNG-irisin are actually 20 and 13 kDa, so the bands identified in that study^22^ are too large. Furthermore, they used the same antibody as Boström *et al.*^1^, designated pAb-E here, which was raised against a C-terminal sequence that is not included in irisin. Finally, they analyzed the 32 and 24 kDa bands by mass spectrometry and reported a single tryptic peptide corresponding to the irisin sequence. We did not find any *FNDC5* or irisin signatures in our immune-precipitated bands identified by western blots of human serum. However, we were able to detect irisin spiked into human serum at the predicted size for glycosylated and non-glycosylated irisin.

Mass spectrometry analysis of the ~20 kDa zone cut from SDS-PAGE of human serum detected a peptide corresponding to *FNDC5* or irisin. This is the same peptide identified by Lee et al^22^, although they found it in ~32 and ~24 kDa bands stained with a pAb against C-terminal *FNDC5*. Consequently, our result is the first mass spectrometry identification of an irisin peptide at the correct size, and might be considered as supporting the existence of irisin in human serum. However, none of the tested irisin antibodies stained a band at this size in serum. This underlines that glycosylated irisin has not been detected by western blot before and that none of the ELISA kits used in previous studies have measured irisin levels in serum or plasma of any species.

The birth of the new, promising myokine irisin^1^ has been complicated from the beginning by multiple contradictions and obscurities in the interpretation of western blot data^18^. Numerous subsequent studies relied on ELISAs with commercial antibodies that were not sufficiently validated in biological fluids. This resulted in highly contradictory data concerning the existence of irisin and its role in humans and other species. Our study targeted the basic question behind the controversy – the physical existence of the proposed cleavage product of *FNDC5* in circulation. The key to this was to use western blots to visualize and avoid the false signal from cross-reacting proteins of the polyclonal antibodies, and to use recombinant, non-glycosylated and glycosylated irisin as positive controls. In addition to calling into question all previous studies using commercial ELISAs, we found no evidence for circulating irisin in human or several animal species when examined by western blot with 4 different antibodies and a sensitive detection system. Although we report the first peptide signature size-matched to rG-irisin by HPLC/mass spectrometry in human serum, the apparently low concentration – below the detection limit of the tested antibodies – makes a physiological role for irisin very unlikely. Atherton & Philips^32^ raised the question whether irisin is a “Greek goddess or a Greek myth”. Our results provide experimental evidence for irisin being a myth.

## Methods

### Ethical approval

The human exercise study named MyoGlu adhered to the Declaration of Helsinki and was approved by the National Regional Committee for Medical and Health Research Ethics North, Tromsø, Oslo, Norway. The study was registered with the US National Library of Medicine Clinical Trials registry (NCT01803568). Written informed consent was obtained from all participants prior to any study-related procedure. Equine blood samples were taken in accordance with the local animal ethics committee (Etat de Vaud, Service Vétérinaire, Switzerland).

### Strength and endurance training intervention

Healthy, sedentary men (40–65 years) were recruited in 2 groups; controls with normal weight (23.5 ± 2.0 kg/m^2^) and normal fasting and 2 h serum oral glucose tolerance test (OGTT) levels (n = 13) or overweight (29.0 ± 2.4 kg/m^2^) with abnormal glucose metabolism (pre-diabetes group, n = 13). Abnormal glucose metabolism was defined as fasting glucose ≥ 5.6 mmol/L and/or impaired glucose tolerance (2 hours serum OGTT ≥ 7.8 mmol/L). The participants underwent 2 endurance bicycle sessions (60 min) and 2 whole-body strength-training sessions (60 min) per week for 12 weeks. A 45 min bicycle session at 70% of VO_2_max was performed before and after the 12 week training period as an acute work challenge^13^.

### Blood and tissue sampling

Blood and muscle samples were taken before, directly after, and 2 h after the 70% of VO_2_max bicycle test, before as well as after 12 weeks of training. Blood samples were taken by standard antecubital venous puncture. Biopsies from *m. vastus lateralis* were immediately rinsed in phosphate buffered saline (PBS), dissected free of blood, before they were transferred to RNA-later (Qiagen, Limburg, Netherlands; overnight at 4°C) and finally transferred to −80°C. Serum was stored at −80°C until analysis.

### Animal serum and plasma samples

Samples for species comparison were obtained from commercial suppliers (donkey, goat, rabbit), from previous studies in swine and wild boar^33^, and from our own investigations in cattle^24^ and mice^23^. These samples were not further characterized and represented the respective species only.

Serum of a horse that underwent a moderate physical exercise program (45 min with intervals of walking, trotting and 3 times 1500 m at a speed of 8.5, 8.4, and 10.3 m/s) was sampled in the Swiss National Stud in Avenches (Switzerland) immediately and 30 min post exercise. Further samples were taken from 5 horses immediately or 60 min after participation in a 160 km endurance race (Ramboulliet, France). One horse with confirmed metabolic syndrome was sampled at the Swiss Institute of Equine Medicine (ISME), Vetsuisse Faculty, University of Berne.

### Expression and purification of recombinant, non-glycosylated and glycosylated irisin

For convenient his-tag removal, the bacterial irisin expression construct^34^ was modified by inserting the 3C protease cleavage sequence (LEVLFQGP) in the Nde I site. This modification dramatically reduced protein solubility when it was expressed in *E. coli* BL21 (DE3) at 37°C, so the his-3C-irisin construct was expressed in *E. coli* C41 (DE3)^35^ at room temperature. Expressed proteins were purified with a cobalt column (TALON; Clontech, Mountain View, USA) using standard protocols. Purified proteins eluted with imidazole from the column were dialyzed against phosphate buffered saline (PBS, pH 7.2) in the presence of his-tagged 3C protease at 4°C. After dialysis, samples were run through the cobalt column again to remove uncleaved his-tagged irisin, cleaved his-tagged portions and his-tagged 3C protease. Non his-tagged irisin was recovered in the flow through fraction from the cobalt column. The amino acid sequence of rNG-irisin (no his-tag) is gphmSPSAPVNVTVRHLKANSAVVSWDVLEDEVVIGFAISQQKKDVRMLRFIQEVNTTTRSCALWDLEEDTEYIVHVQAISIQGQSPASEPVLFKTPREAEKMASKNKDEVTMKE (the sequence in lowercase is derived from the vector). Recombinant G-irisin was produced in mammalian cells^34^. The irisin expression construct (irisin/pHLSec2) was transfected into HEK293 cells and the conditioned medium was collected after 6-7 days of transfection. Secreted protein was purified with the cobalt column and the C-terminal his-tag was not removed. The amino acid sequence of rG-irisin is egsADSPSAPVNVTVRHLKANSAVVSWDVLEDEVVIGFAISQQKKDVRMLRFIQEVNTTTRSCALWDLEEDTEYIVHVQAISIQGQSPASEPVLFKTPREAEKMASKNKDEVTMKEefhhhhhhh. Glycosylation was verified by a molecular weight shift in SDS-PAGE after PNGase F (NEB, Ipswich, USA) treatment. The concentrations of purified proteins were estimated from the absorbance at 280 nm using the extinction coefficients of the proteins determined by the Protean computer program (DNAstar, Madison, USA).

In one experiment synthetic irisin (Catalog # 067-16; Phoenix Europe, Karlsruhe, Germany) was used as an additional control.

### Antibodies

The following antibodies were used in this study: pAb-A, rabbit polyclonal antibody (pAb) to irisin (Phoenix Europe, Karlsruhe, Germany); pAb-B, rabbit pAb to irisin (Cayman Chemicals, Ann Arbor, USA); pAb-C, rabbit pAb to irisin (AdipoGen, Liestal, Switzerland); pAb-D, rabbit pAb to irisin (Phoenix Europe); and pAb-E, rabbit pAb to *FNDC5* (C-terminal; BioCat, Heidelberg, Germany). Antibodies pAb-A and C were raised against full length rNG-irisin and pAb-B and D against the full-length synthetic irisin peptide. Antibody pAb-E was generated against the C-terminus of *FNDC5*, which is not part of the irisin peptide. Details on the antibodies are given in Table 1.

### Western immunoblotting

For dilution series, rNG-irisin was diluted into either PBS or bovine plasma. Irisin stock solution (1 μg/μL in PBS) was first diluted 1:100 in bovine plasma resulting in minimal dilution of plasma proteins. This recombinant NG-irisin solution was further diluted in bovine plasma to final concentrations of 10, 2, 1, 0.5, 0.25, 0.125, 0.0625, and 0.03125 ng/μL. Two microliters of each sample were then mixed 1:2 with loading buffer (2× Laemmli sample buffer with 10% β-mercaptoethanol), denatured at 95°C for 5 min and separated on Criterion TGX 12% polyacrylamide gels (BioRad). Absolute amounts of rNG-irisin analyzed per lane were consequently between 20 ng and 0.0625 ng. Each 1 μL of native murine and human serum samples and 20 μg murine muscle protein extract were treated as described above and electrophoresed on the same gel.

For species comparison, 1 μL of serum or plasma from the following species was used: horse, cattle (bull, cow, calf), pig (domestic and wild), donkey, goat, rabbit, mouse, and human. Samples were analyzed under the same conditions as described above. Recombinant NG-irisin (2 μL from 1 ng/μL stock solution) and rG-irisin (1 μL from 10 ng/μL stock solution) were used as positive controls.

Proteins were transferred to a polyvinylidene difluoride (PVDF) membrane (Trans-Blot Turbo transfer pack, Bio-Rad, Munich, Germany) using a semi-dry blotter (Trans-Blot, Bio-Rad). Equal loading of the gels and proper transfer of the proteins to the membranes were verified by Coomassie Blue staining according to standard procedures. Membranes were blocked for 1 h in either 1× Roti-Block (Roth), for detection of irisin (pAb-A, B, C and D), or in 5% non-fat dry milk in Tris-buffered saline (TBS) for detection of *FNDC5* (pAb-E). Membranes were incubated with primary antibodies (pAb-A: diluted 1:3,000, pAb-B: 1:500, pAb-C: 1:2,000, and pAb-E 1:1,000) overnight at 4°C.

After washing, membranes were incubated with horseradish peroxidase-conjugated secondary antibody (rabbit IgG TrueBlot, 1:25,000; 18–8,816, eBioscience, Frankfurt, Germany). Antibody label was detected with chemiluminescence substrate (Super Signal West Femto, Thermo Fisher Scientific, Bonn, Germany) and a Chemocam HR-16 imager (INTAS, Göttingen, Germany). LabImage 1D software (Kapelan Bio-Imaging, Leipzig, Germany) was used to quantify the volumes of specific bands.

### Analysis of serum irisin

Concentrations of irisin were measured in duplicates in human serum using enzyme-linked immuno-sorbent assays (ELISA [based on pAb-C]; Table 1) according to the manufacturer's protocol. Optical density was determined using a microplate reader (Termo Scientific Multiscan EX, Vantaa, Finland) set to 450 nm. Data from a previous measurement of irisin in plasma samples of the same individuals^13^ with a different ELISA (based on pAb-A; Table 1) were used for comparison. Letters assigned to the ELISAs correspond to the letters designated to the antibodies.

### Immuno-precipitation and SDS-PAGE of serum samples with added irisin

MagnaBind magnetic beads (Pierce, Thermo Fisher Scientific, Bonn, Germany), pre-coated with anti-rabbit IgG antibody, were incubated with anti-irisin antibody (Adipogen) for 60 min under permanent agitation (Dynabeads MX Mixer, Life Technologies, Darmstadt, Germany). Separation steps were performed using InviMag (STRATEC, Berlin, Germany) magnetic separator. Binding of antibodies to the beads was made permanently according to standard protocols by using cross-linking buffer (25 mM DMP) at room temperature for 45 min. To stop the reaction, blocking buffer (0.1 M Ethanolamine in PBS, pH 8.2) was used. Unbound antibody was eluted from the beads with elution buffer (1 M glycine-HCL, pH 3). Serum (500 μl) was denatured for 5 min at 95°C and added to the antibody coated beads. Incubation was performed overnight at 4°C under permanent agitation. After several washes, proteins were eluted from beads with elution buffer (1 M glycine-HCL, pH 3). To optimize the protocol for subsequent mass spectrometry, the elution buffer was changed (8 M urea, 20 mM Tris pH 7.5, 100 mM NaCl) according to standard protocols (abcam, Cambridge, U.K.) in a repeated trial.

Eluted proteins were separated on a 15% polyacrylamide gel and stained with QuickCoomassie (Serva, Heidelberg, Germany) according to manufacturer's instructions. Bands of interest were cut out of the gel and submitted to analysis by mass spectrometry. Furthermore, human serum samples were albumin-depleted with Aurum Affi-Gel Blue mini columns (Bio-Rad) as described elsewhere^24^. One hundred or 500 ng of either rNG- or rG-irisin were then added to the samples. The samples (20 μg total protein) were separated by SDS-PAGE (15%) and stained with QuickCoomassie (Serva). Serum samples without addition of rNG-/rG-irisin were also analyzed. Recombinant NG-/rG-irisn (500 ng) diluted in PBS were used for size comparison. Regions of interest were cut out of the gel and analyzed by mass spectrometry.

### Protein identification by mass spectrometry

The Coomassie stained gel bands were in-gel digested using trypsin. The generated peptides were purified using μ-C18 ZipTips (Millipore, Oslo, Norway), dried in a SpeedVac, dissolved in 10 μL 1% formic acid, 2.5% acetonitrile in water. Half of the volume was injected into a nano-UHPLC system (Ultimate 3000 RSLC, Dionex, Sunnyvale, CA, USA) coupled to an ESI-ion trap/Orbitrap (LTQ Orbitrap XL, Thermo Scientific, Bremen, Germany) mass spectrometer. For peptide separation, an Acclaim PepMap 100 column (50 cm × 75 μm) packed with 100 Å C18 3 μm particles (Dionex; Nerliens Meszansky, Oslo, Norway) was used with a flow rate of 300 nL/min and a solvent gradient of 3% B to 35% B for 45 min. Solvent A was 0.1% formic acid and solvent B was 0.1% formic acid/90% acetonitrile. Survey full scan MS spectra (from m/z 300 to 2000) were acquired in the Orbitrap with the resolution R = 60,000 at m/z 400 after accumulation to a target of 1,000,000 charges in the LTQ. The method allowed sequential isolation of up to the 7 most intense ions for fragmentation on the linear ion trap using collision induced dissociation (CID) at a target value of 10,000 charges. Target ions already selected for MS/MS were dynamically excluded for 60 sec. Data were acquired using Xcalibur v2.5.5 (Thermo Scientific) and raw files were processed to generate peak list in Mascot generic format (*.mgf) using ProteoWizard release version 3.0.331^36^. Database searches were performed using Mascot in-house version 2.4. to search the SwissProt database assuming the digestion enzyme trypsin, at maximum one missed cleavage, fragment ion mass tolerance of 0.60 Da, parent ion tolerance of 10 ppm and oxidation of methionines, and acetylation of the protein N-terminus as variable modifications. Scaffold (version Scaffold_4.3.4, Proteome Software, Portland, USA) was used to validate MS/MS based peptide and protein identifications. Peptide identifications were accepted if they could be established at greater than 95% probability by the Scaffold Local FDR algorithm. Protein identifications were accepted when they could be established at greater than 99% probability and contained at least 2 identified peptides.

### High throughput mRNA sequencing and differential expression analysis

Skeletal muscle biopsies sampled before the start of acute exercise at baseline and after 12 weeks of training were deep-sequenced in 6 batches using the Illumina HiSeq 2000 system (Illumina, San Diego, USA) with multiplexing at the Norwegian Sequencing Centre, University of Oslo. Samples in different groups and time points were multiplexed between batches to avoid batch effects. Illumina HiSeq RTA (real-time analysis) v1.17.21.3 (Illumina) was used for real-time analysis during the sequencing. Reads passing Illumina's recommended parameters were demultiplexed using CASAVA v1.8.2 (Illumina). For prealignment quality checks we used the software FastQC v0.10.1. (Babraham Bioinformatics, Cambridge, UK). To avoid low quality data negatively influencing downstream analysis, reads were trimmed on the 3′-end and only the first 51 bp from the 5′-end of each read were kept for further analysis. The mean library size was ~44 million unstranded 51 bp single-ended reads with no difference between groups or time points. Base composition in bases 1–12 showed patterns typical for RNA-seq and bases 13-51 were evenly distributed. All base positions were of high quality (Phred score >30). Alignment of sequenced cDNA reads to the RefSeqGene database as transcriptome reference and quantitative analysis of the transcripts was done as described by Li et al. (2014)^37^.

### Statistical analysis

ELISA and western blot data were analyzed with ANOVA using PROC MIXED (SAS Institute, Cary, USA) with the following factors included in the model: fixed factor group and repeated factor treatment (with unstructured covariance structure) and corresponding interaction, plate and blot were included as random effect, respectively. Differences between means were considered significant if p < 0.05. Pearson correlation coefficients were calculated among quantitative data from ELISAs and western blots.

## Author Contributions

E.A., F.N., B.T., T.O., T.H., L.S., S.L., S.T. and J.B. performed the experiments, provided samples, materials and data, analyzed the data, and drafted parts of the manuscript. S.M., H.P.E. and C.A.D. conceived the study. S.M. coordinated the experiments and S.M. and H.P.E. wrote the manuscript.

## Supplementary Material

Supplementary Information Supplementary Information

## Figures and Tables

**Table 1:** Polyclonal irisin antibodies used in this and related ELISA kits

Designation (this study)	Manufacturer/Vendor (Cat. #)	Antigen (epitope)^1^	Manufacturer (ELISA #)	Range of ELISA^2^	Remarks
pAb-A	Aviscera/Phoenix (G-067-52)	recombinant irisin (aa 33–142)	Phoenix (EK-067-52)	0.3–205 ng/mL	antibody discontinued; ELISA replaced by EK-067-29
pAb-B	Cayman (14625)	synthetic irisin (aa 33–142)	-	-	no ELISA available
pAb-C	Adipogen (A-25B-0027)	recombinant irisin (aa 33–142)	Adipogen (AG-45A-0046EK)	1.0–5,000 ng/mL	antibody is epitope affinity purified
pAb-D	Phoenix (G-067-16)	synthetic irisin (aa 33–142)	Phoenix (EK-067-16)	0.1–1,000 ng/mL	ELISA not used in this study
pAb-E	BioVision/BioCat(AP8746b-AB)	C-terminus of *FNDC5* (aa 149–178)	-	-	no ELISA available antibody is epitope affinity purified

^1^refers to GenPept accession number NP_715637.2.

^2^data of manufacturer/vendor.

## References

[b1] Boström P. *et al.* A PGC1-α-dependent myokine that drives brown-fat-like development of white fat and thermogenesis. Nature 481, 463–468 (2012). 22237023 10.1038/nature10777 PMC3522098

[b2] Ivanov I. P., Firth A. E., Michel A. M., Atkins J. F. & Baranov P. V. Identification of evolutionarily conserved non-AUG-initiated N-terminal extensions in human coding sequences. Nucleic Acids Res. 39, 4220–4234 (2011). 21266472 10.1093/nar/gkr007 PMC3105428

[b3] Raschke S. *et al.* Evidence against a beneficial effect of irisin in humans. PLOS ONE 8, e73680 (2013). 24040023 10.1371/journal.pone.0073680 PMC3770677

[b4] Timmons J. A., Baar K., Davidsen P. K. & Atherton P. J. Is irisin a human exercise gene? Nature 488, E9–10 (2012). 22932392 10.1038/nature11364

[b5] Huh J. Y. *et al.* FNDC5 and irisin in humans: I. Predictors of circulating concentrations in serum and plasma and II. mRNA expression and circulating concentrations in response to weight loss and exercise. Metabolism 61, 1725–1738 (2012). 23018146 10.1016/j.metabol.2012.09.002 PMC3614417

[b6] Kraemer R. R., Shockett P., Webb N. D., Shah U. & Castracane V. D. A transient elevated irisin blood concentration in response to prolonged, moderate aerobic exercise in young men and women. Horm. Metab. Res. 46, 150–154 (2014). 24062088 10.1055/s-0033-1355381

[b7] Tsuchiya Y. *et al.* High-Intensity exercise causes greater irisin response compared with low-intensity exercise under similar energy consumption. Tohoku J. Exp. Med. 233, 135–140 (2014). 24910199 10.1620/tjem.233.135

[b8] Anastasilakis A. D. *et al.* Circulating irisin in healthy, young individuals: Day-night rhythm, effects of food intake and exercise, and associations with gender, physical activity, diet and body composition. J. Clin. Endocrinol. Metab. 99, 3247–3255 (2014). 24915120 10.1210/jc.2014-1367

[b9] Daskalopoulou S. S. *et al.* Plasma irisin levels progressively increase in response to increasing exercise workloads in young, healthy, active subjects. Eur. J. Endocrinol. 171, 343–352 (2014). 24920292 10.1530/EJE-14-0204

[b10] Pekkala S. *et al.* Are skeletal muscle FNDC5 gene expression and irisin release regulated by exercise and related to health? J. Physiol. 591, 5393–5400 (2013). 24000180 10.1113/jphysiol.2013.263707 PMC3936375

[b11] Hecksteden A. *et al.* Irisin and exercise training in humans – Results from a randomized controlled training trial. BMC Med. 11, 235 (2013). 24191966 10.1186/1741-7015-11-235 PMC4228275

[b12] Kurdiova T. *et al.* Are skeletal muscle & adipose tissue Fndc5 gene expression and irisin release affected by obesity, diabetes and exercise? In vivo & in vitro studies. J. Physiol. 592, 1091–1107 (2014). 24297848 10.1113/jphysiol.2013.264655 PMC3948565

[b13] Norheim F. *et al.* The effects of acute and chronic exercise on PGC-1α, irisin and browning of subcutaneous adipose tissue in human. FEBS J. 281, 739–749 (2014). 24237962 10.1111/febs.12619

[b14] Hofmann T. *et al.* Irisin levels are not affected by physical activity in patients with anorexia nervosa. Front. Endocrinol. (Lausanne) 4, 202 (2014). 24432013 10.3389/fendo.2013.00202 PMC3880939

[b15] Scharhag-Rosenberger F. *et al.* Irisin does not mediate resistance training-induced alterations in RMR. Med. Sci. Sports Exerc. 46, 1736–1743 (2014). 24566753 10.1249/MSS.0000000000000286

[b16] Alvehus M., Boman N., Söderlund K., Svensson M. B. & Burén J. Metabolic adaptations in skeletal muscle, adipose tissue, and whole-body oxidative capacity in response to resistance training. Eur. J. Appl. Physiol. 114, 1463–1471 (2014). 24711079 10.1007/s00421-014-2879-9

[b17] Ellefsen S. *et al.* Irisin and FNDC5: effects of 12-week strength training, and relations to muscle phenotype and body mass composition in untrained women. Eur. J. Appl. Physiol. 114, 1875–1888 (2014). 24906447 10.1007/s00421-014-2922-x

[b18] Erickson H. P. Irisin and FNDC5 in retrospect: An exercise hormone or a transmembrane receptor? Adipocyte 2, 289–293 (2013). 24052909 10.4161/adip.26082 PMC3774709

[b19] Sharma N., Castorena C. M. & Cartee G. D. Greater insulin sensitivity in calorie restricted rats occurs with unaltered circulating levels of several important myokines and cytokines. Nutr. Metab. (Lond) 9, 90 (2013). 23067400 10.1186/1743-7075-9-90 PMC3541154

[b20] Wen M. S., Wang C. Y., Lin S. L. & Hun K. C. Decrease in irisin in patients with chronic kidney disease. PLOS ONE 8, e64025 (2013). 23667695 10.1371/journal.pone.0064025 PMC3646802

[b21] Roca-Rivada A. *et al.* FNDC5/Irisin is not only a myokine but also an adipokine. PLOS ONE 8, e60563 (2013). 23593248 10.1371/journal.pone.0060563 PMC3623960

[b22] Lee P. *et al.* Irisin and FGF21 are cold-induced endocrine activators of brown fat function in humans. Cell Metab. 19, 302–309 (2014). 24506871 10.1016/j.cmet.2013.12.017 PMC7647184

[b23] Brenmoehl J. *et al.* Irisin is elevated in skeletal muscle and serum of mice immediately after acute exercise. Int. J. Biol. Sci. 10, 338–349 (2014). 24644429 10.7150/ijbs.7972 PMC3957089

[b24] Komolka K. *et al.* Locus characterization and gene expression of bovine FNDC5: Is the myokine irisin relevant in cattle? PLOS ONE 9, e88060 (2014). 24498244 10.1371/journal.pone.0088060 PMC3909329

[b25] Sanchis-Gomar F., Alis R., Pareja-Galeano H., Romagnoli M. & Perez-Quilis C. Inconsistency in Circulating Irisin Levels: What is Really Happening? Horm. Metab. Res. 46, 591–596 (2014). 24459033 10.1055/s-0033-1363283

[b26] Elsen M., Raschke S. & Eckel J. Browning of white fat: does irisin play a role in humans? J. Endocrinol. 222, R25–38 (2014). 24781257 10.1530/JOE-14-0189

[b27] Wrann C. D. *et al.* Exercise induces hippocampal BDNF through a PGC-1α/FNDC5 pathway. Cell Metab. 18, 649–659 (2013). 24120943 10.1016/j.cmet.2013.09.008 PMC3980968

[b28] Elsen M., Raschke S., Sommerfeld M., Gassenhuber H. & Eckel J. Comment on Wu and Spiegelman. Irisin ERKs the Fat. Diabetes 63, 381–383 (2014). Diabetes 63, e16 (2014). 25146477 10.2337/db14-0776

[b29] Spiegelman B. M. & Wrann C. Response to comment on Wu and Spiegelman. Irisin ERKs the Fat. Diabetes 63, 381–383 (2014). Diabetes 63, e17 (2014). 25146478 10.2337/db14-0898

[b30] Choi H. Y. *et al.* Implication of circulating irisin levels with brown adipose tissue and sarcopenia in humans. J. Clin. Endocrinol. Metab. 99, 2778–2785 (2014). 24780049 10.1210/jc.2014-1195

[b31] Zhang Y. *et al.* Irisin stimulates browning of white adipocytes through mitogen-activated protein kinase p38 MAP kinase and ERK MAP kinase signaling. Diabetes 63, 514–525 (2014). 24150604 10.2337/db13-1106 PMC13117908

[b32] Atherton P. J. & Phillips B. E. Greek goddess or Greek myth: the effects of exercise on irisin/FNDC5 in humans. J. Physiol. 591, 5267–5268 (2013). 24187077 10.1113/jphysiol.2013.265371 PMC3936363

[b33] Lösel D., Franke A. & Kalbe C. Comparison of different skeletal muscles from growing domestic pigs and wild boars. Arch. Tierz. 56, 76 (2013).

[b34] Schumacher M. A., Chinnam N., Ohashi T., Shah R. S. & Erickson H. P. The structure of irisin reveals a novel intersubunit β-sheet fibronectin type III (FNIII) dimer: implications for receptor activation. J. Biol. Chem. 288, 33738–33744 (2013). 24114836 10.1074/jbc.M113.516641 PMC3837118

[b35] Miroux B. & Walker J. E. Over-production of proteins in Escherichia coli: mutant hosts that allow synthesis of some membrane proteins and globular proteins at high levels. J. Mol. Biol. 260, 289–298 (1996). 8757792 10.1006/jmbi.1996.0399

[b36] Chambers M. C. *et al.* A cross-platform toolkit for mass spectrometry and proteomics. Nat. Biotechnol. 30, 918–920 (2012). 23051804 10.1038/nbt.2377 PMC3471674

[b37] Li Y. *et al.* Subsarcolemmal lipid droplet responses to a combined endurance and strength exercise intervention. Physiol. Rep. 2, e12187 (2014). 25413318 10.14814/phy2.12187 PMC4255802

