# Supplementary material for: Irisin – a myth rather than an exercise-inducible myokine
Source: Sci Rep. 2015 Mar 9;5:8889. doi: 10.1038/srep08889 (PMC4352853; doi:10.1038/srep08889)
Supplement: Supplementary Information [file srep08889-s1.pdf]

Irisin – a myth rather than an exercise-inducible myokine

Elke Albrecht, Frode Norheim, Bernd Thiede, Torgeir Holen, Tomoo Ohashi, Lisa Schering, Sindre Lee, Julia Brenmoehl, Selina Thomas, Christian A. Drevon, Harold P. Erickson, Steffen Maak

Supplementary figures

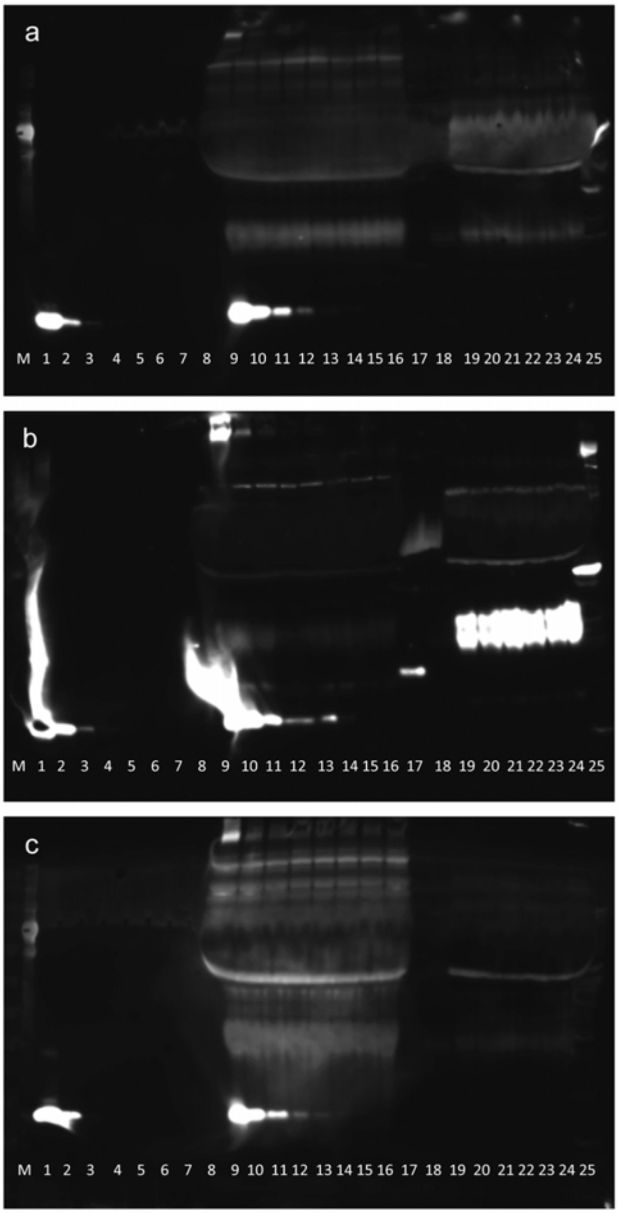

Supplementary Figure 1. Western blot of dilution series of rNG-irisin. Recombinant NG-irisin was diluted in PBS (lanes 1-8; absolute amounts 20, 4, 2, 1, 0.5, 0.25, 0.125, 0.0625 ng irisin/lane) or bovine plasma (lanes 9-16; irisin amounts as in PBS). Mouse serum is shown in lanes 17 and 18, human serum in lanes 19-24, and mouse muscle protein in lane 25. **(a-c)** Detection with pAb-B **(a)**, pAb-C **(b)**, and pAb-D **(c)**. Trueblot rabbit HRP was used as detection system. Original images were taken after 10 min of exposure.

Linearity of detection of irisin was given for all antibodies in the range between 4 ng/lane and 0.125 ng/lane (R^2 = 0.9467 for pAb-B; R^2 = 0.9465 for pAb-C, and R^2 = 0.9715 for pAb-D).

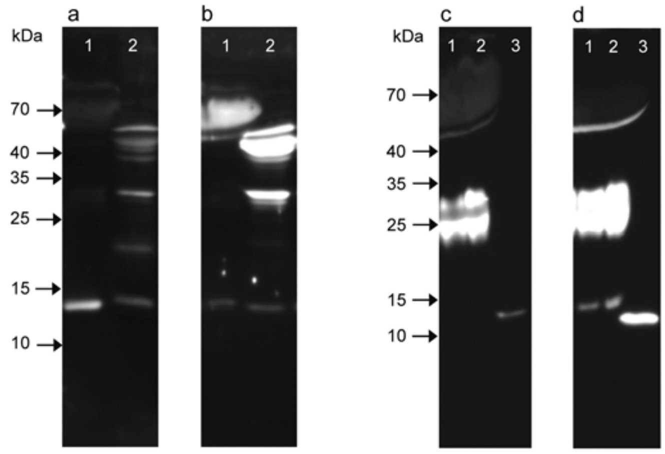

Supplementary Figure 2. Lot-to-lot variability of irisin antibodies. (a, b) Two lots of pAb-A were used to detect irisin in murine serum (lane 1) and muscle (lane 2; Lot # 01478 **(a)** vs. Lot # 01545 **(b)**). **(c, d)** Two lots of pAb-C were used to stain human serum (lanes 1-2) and rNG-irisin as positive control (lane 3; Lot # A25071305 **(c)** vs. Lot # A25481312 **(d)**).

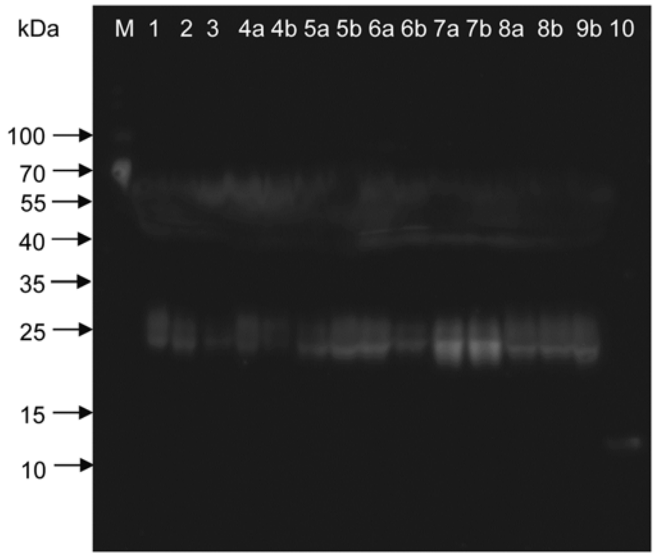

Supplementary Figure 3. Western blot of equine serum with pAb-C. Serum samples of three horses with metabolic diseases (lanes 1-3), of a horse immediately (lane 4a) and 30 min after (lane 4b) 45 min of moderate exercise. Horses (lanes 5-9) were sampled 10-30 min after participation in a 160 km endurance race (lanes a) and 60 min after the first sample (lanes b). Recombinant NG-irisin was included as positive control (lane 10). M: Molecular weight marker. The image was taken after 30 min exposure.

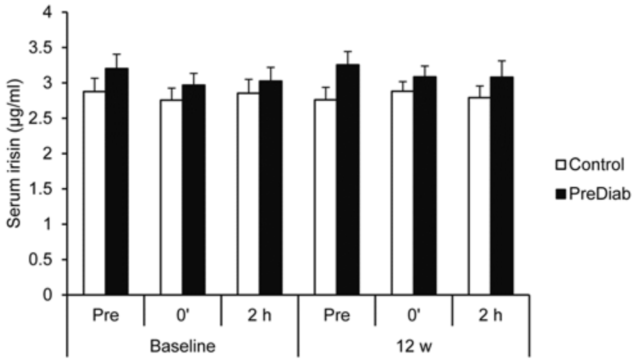

Supplementary Figure 4. Circulating irisin in response to acute exercise (0 and 2 h) or to 12 weeks of training (12 w). Changes in serum concentrations of irisin in healthy controls (white bars) and pre-diabetes subjects (black bars) in response to acute exercise (0 and 2 h) and in response to 12 w of training (n = 13 each). Plasma samples were obtained before (Pre), immediately after (0'), and 2 h post-exercise (2 h) of 45 min ergometer cycling at baseline and after 12 w of training. Bars depict means ± SEM. Student's t-test was used for single comparisons.
